# The effect of erenumab on brain network function in episodic migraine patients: a randomized, placebo-controlled clinical trial (RESET BRAIN)

**DOI:** 10.1007/s00415-023-11879-9

**Published:** 2023-08-08

**Authors:** Massimo Filippi, Roberta Messina, Marta Bartezaghi, Ilaria Cetta, Bruno Colombo, Licia Grazzi, Daniele Martinelli, Raffaele Ornello, Anna Pichiecchio, Debora Raimondi, Antonio Russo, Simona Sacco, Alessandra Splendiani, Cristina Tassorelli, Renato Turrini, Paola Valsasina, Maria Assunta Rocca, RESET BRAIN Study Group, Federico Bruno, Angela Campanella, Valeria Caponnetto, Luca Dall’Occhio, Marcello Silvestro, Roberto Vuotto

**Affiliations:** 1 Neuroimaging Research Unit, Division of Neuroscience, IRCCS San Raffaele Scientific Institute, Via Olgettina, 60, 20132 Milan, Italy; 2 Neurology Unit, IRCCS San Raffaele Scientific Institute, Milan, Italy; 3 Neurorehabilitation Unit, IRCCS San Raffaele Scientific Institute, Milan, Italy; 4 Neurophysiology Service, IRCCS San Raffaele Scientific Institute, Milan, Italy; 5 Vita-Salute San Raffaele University, Milan, Italy; 6 Novartis Farma, Milano, Italy; 7 Neuroalgology Unit, Headache Center Fondazione IRCCS Istituto Neurologico “Carlo Besta”, Milan, Italy; 8 Headache Science and Rehabilitation Center, IRCCS Mondino Foundation, Pavia, Italy; 9 Department of Brain and Behavioral Sciences, University of Pavia, Pavia, Italy; 10 Department Biotechnological and Applied Clinical Sciences, University of L’Aquila, L’Aquila, Italy; 11 Neuroradiology Department, Advanced Imaging and Radiomics Center, IRCCS Mondino Foundation, Pavia, Italy; 12 Department of Advanced Medical and Surgical Sciences (DAMSS), Headache Center, University of Campania “Luigi Vanvitelli”, Naples, Italy

**Keywords:** Headache, Prevention, Therapy, Neuroimaging

## Abstract

**Background:**

We aimed to explore whether erenumab, a monoclonal antibody targeting the calcitonin gene-related peptide receptor, could exert a central effect on brain network function in migraine, and investigate the persistence of such an effect following treatment discontinuation.

**Methods:**

This was a randomized, double-blind, placebo-controlled, multicenter trial with a crossover design performed in adult episodic migraine patients with previous treatment failure. Patients were randomized (1:1) to 12 weeks of erenumab 140 mg or placebo, followed by a 12-week crossover. Resting state (RS) functional connectivity (FC) changes of brain networks involved in migraine were investigated using a seed-based correlation approach.

**Results:**

Sixty-one patients were randomized to treatment. In each treatment sequence, 27 patients completed the visit at week 12. Forty-four enrolled patients, 22 in each treatment sequence, completed the study procedures with no major protocol violations. We observed a carry-over effect of erenumab during the placebo treatment and therefore data analysis was performed as a parallel comparison of erenumab vs placebo of the first 12 weeks of treatment. From baseline to week 12, compared to placebo, patients receiving erenumab showed RS FC changes within the cerebellar, thalamic and periaqueductal gray matter networks, significantly associated with clinical improvement. Compared to non-responders, patients achieving a 50% reduction in migraine days had distinct patterns of thalamic and visual network RS FC. Brain RS FC changes reversed when erenumab was stopped. A lower baseline RS FC of the pontine network identified patients responding to erenumab*.*

**Conclusion:**

Erenumab modulates RS FC of networks involved in migraine pathophysiology. In line with clinical response, erenumab-induced brain RS FC changes tend to reverse when treatment is stopped.

**Supplementary Information:**

The online version contains supplementary material available at 10.1007/s00415-023-11879-9.

## Introduction

Migraine is a common and highly disabling neurological disease [1]. Increased recognition of the huge personal and social impact of migraine has increased interest in the development of new treatments. Based on evidence supporting a key role of calcitonin gene-related peptide (CGRP) in migraine neurobiology, new migraine-specific preventive drugs targeting the CGRP pathway have been developed [2]. Erenumab is a monoclonal antibody (mAb) targeting the CGRP receptor. Previous randomized controlled trials (RCTs) showed the efficacy, safety and tolerability of erenumab in episodic and chronic migraine [3–5]. Erenumab is believed to exert its antimigraine effect in the periphery, more precisely at the trigeminovascular level. This belief is sustained by the large size of anti-CGRP mAbs and their poor blood–brain barrier (BBB) penetrability (1:1000 ratio) [6]. In this context, modulation of the trigeminal ganglion and fibers may limit the recruitment of pain signaling at the meningeal tissues and inhibit pain transmission at the trigeminal ganglion and nucleus [2]. On the other hand, the rich expression of CGRP and CGRP receptors in numerous brain regions implicated in migraine pathophysiology, including the trigeminal cervical complex, thalamus, hypothalamus and brainstem, may support a possible central effect of the small amount of mAbs penetrating the BBB [7, 8], especially when considering that the choroid plexus is likely to allow large antibodies entering the cerebrospinal fluid (CSF) [9].

The application of advanced magnetic resonance imaging (MRI) techniques has markedly enhanced the understanding of migraine [10]. Two observational functional MRI (fMRI) studies showed that two-week treatment with anti-CGRP mAbs changed the activity of the thalamus, cerebellum, insular and somatosensory cortices in migraine patients during trigeminal stimulation [11, 12]. A recent observational study revealed that clinical response to eight weeks of erenumab was associated with fMRI changes involving the hypothalamus, amygdala, periaqueductal gray (PAG) and parieto-temporal brain areas [13]. Overall, these studies demonstrated brain fMRI changes in migraine patients treated with mAbs targeting the CGRP pathway [11–13]. However, fMRI studies examining central effects of anti-CGRP mAbs after 3 months of treatment and controlling for placebo effects are missing. Moreover, whether treatment-related functional brain changes persist or reverse when anti-CGRP mAbs are discontinued have never been investigated.

Here, using a blinded, placebo-controlled design, we primarily aimed to confirm central modifications mediated by erenumab and resting state (RS) functional connectivity (FC) patterns of responders and non-responders after 12 weeks of therapy. Secondary and exploratory aims of the study were to: (1) explore whether central modulation of RS FC contributes to explain the therapeutic effect of erenumab; (2) identify imaging biomarkers of positive response to erenumab; and (3) investigate whether erenumab could lead to sustained central effects after 3-month treatment discontinuation.

Gathering data on the effects of erenumab administration, their persistence and their relation with clinical response is crucial for a better understanding of the mechanism of action of this drug within migraine neurobiology.

## Materials and methods

### Study design and participants

This was a randomized, double-blind, placebo-controlled, crossover, phase 4 trial performed in five Italian Headache Centers from 30 July 2019 to 5 July 2021.

**Fig. 1:**
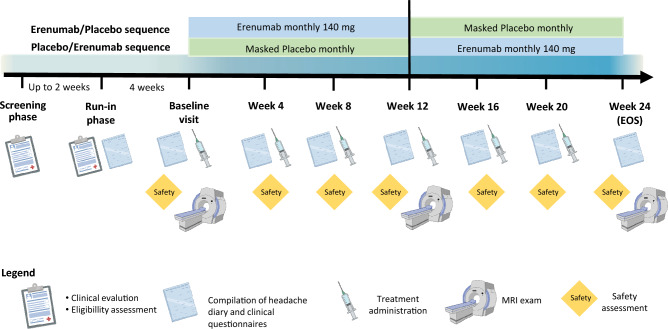
Schematic description of study design and study procedures. *EOS* end of study

The study included a screening (6 weeks), run-in (4 weeks) and a treatment phase (24 weeks). After run-in, patients were randomized to 12-weeks of subcutaneous erenumab 140 mg or masked placebo, followed by 12-weeks of the other treatment (Fig. 1). All patients met diagnostic criteria of episodic migraine according to the International Classification of Headache Disorders (ICHD-3) [14].

Eligible patients had to be older than 18 years, have ≥ 4 and < 15 migraine days/month and have failed two or more previous migraine preventives. See supplementary methods for detailed inclusion/exclusion criteria.

### Randomisation and masking

Patients were randomized in a 1:1 ratio to erenumab or placebo using randomization and medication lists created by the Contract Research Organization’s statistician through a validated SAS program. Randomization was stratified by center. Patients, investigators and the clinical trial team remained blinded to treatment allocation. Erenumab and placebo were supplied in two 70 mg/1 mL pre-filled syringes. Treatments were identical in packaging, labeling and appearance.

### Procedures

During the 24-week treatment phase, patients received erenumab 140 mg or placebo every 4 weeks. The treatment phase comprised a baseline visit and follow-up visits at week 4, 8, 12, 16 and 20. An end of study (EOS) visit was performed at week 24. At baseline visit, patients were randomized and received erenumab or placebo treatment which was continued for the following 12 weeks. At week 12, patients were switched to the other treatment (from erenumab to placebo or vice versa).

During screening, patients’ underwent electrocardiogram, neurological examination, physical examination and a detailed clinical history, including prior headache characteristics, average headache and migraine days of the 3 months preceding study entry and prior migraine preventives failure history. From the run-in phase to the end of the study, patients were asked to complete a paper headache diary reporting the monthly migraine days (MMD), migraine attack duration, monthly headache days (MHD), monthly number of days with use of acute treatments (MAT), pain severity according the numerical rating scale (NRS) [15], monthly number of days with nausea, photophobia or phonophobia and the presence of aura. Definition of a migraine day and duration of migraine attack is reported in Supplementary methods. At run-in, baseline, follow-up and EOS visits, depression and anxiety scores were evaluated using the Hospital Anxiety and Depression Scales (HADS-A and HADS-D) [16] and headache impact was investigated using the Headache Impact Test-6 (HIT-6) [17]. The presence of cutaneous allodynia was investigated using the 12-item Allodynia Symptom Checklist (ASC-12) at baseline, after 12 and 24 weeks of treatment.

At baseline, week 12 and week 24, all participants underwent a brain MRI including RS functional MRI (fMRI), fluid-attenuated inversion recovery, 3D T1-weighted and T2-weighted images, according to a prespecified protocol provided by the central reading facility (Neuroimaging Research Unit, IRCCS San Raffaele Scientific Institute, Milan). The baseline MRI was performed within three days prior to receiving the first dose of study treatment and at week 12 the MRI was performed within three days prior to administration of the fourth dose of treatment in order to explore the effect of 3-month of erenumab. To avoid measuring imaging changes related to acute migraine symptoms, all brain MRI were performed in a migraine/headache-free phase and at least 24 h after the last migraine attack.

Vital signs, clinical laboratory parameters and occurrence of adverse events (AE) were investigated during the entire study. All patients who received at least one dose of study treatment were included in the Safety population.

### Outcomes

Primary endpoints were RS FC changes between erenumab and placebo, as well as between clinical response groups within the two treatment groups. A clinical response was defined as the achievement of at least a 50% reduction of MMD [18]. Secondary endpoints were correlations between brain network RS FC changes and changes in patients’ clinical characteristics. Additional secondary endpoints were MRI predictors of clinical treatment response, efficacy and safety endpoints. Exploratory endpoints were brain network RS FC changes detected in the erenumab group from baseline to week 12, compared to those detected from week 12 to week 24.

### MRI acquisition protocol

Using 3.0 T scanners at all sites, a T2*-weighted single-shot echo planar imaging (EPI) sequence was acquired at all timepoints for RS fMRI (repetition time [TR] = 2000 ms, echo time [TE] = 35 ms, flip angle = 90°, field of view [FOV] = 240 mm^2^; matrix = 64 × 64, 250 sets of 36, 4-mm-thick axial slices). During RS fMRI scanning, subjects were instructed to keep their eyes closed, to remain motionless and not to think anything in particular. The MRI acquisition also included: (a) brain T2-weighted and fluid-attenuated inversion recovery (FLAIR) axial sequences for exclusion of gross brain abnormalities; and (b) brain sagittal 3D T1-weighted. A detailed description of MRI acquisition procedures is provided in the supplementary methods.

### RS fMRI preprocessing

RS fMRI data processing was performed using the CONN toolbox [19]. RS fMRI images were realigned to the mean of each session using a rigid-body transformation to correct for head movements. After rigid registration of realigned images to the 3D T1-weighted scan, reformatted to the axial orientation, RS fMRI images were normalised to the Montreal Neurological Institute space using a non-linear transformation. After detection of outliers (using the ART tool), images were smoothed with a 6-mm^3^ Gaussian filter. For denoising, the first five cerebro-spinal fluid and white matter principal components (segmented from the 3D T1-weighted scan) were used as nuisance covariates in accordance with the anatomical component-based noise correction method (aCompCor) [20]. The six rigid motion parameters and their first temporal derivatives were regressed out from data. Outliers detected by the ART toolbox (if any) and spurious effects from the first two time-points (to maximize magnetic equilibrium) were also regressed out from data. Finally, RS fMRI time series were linearly detrended and band-pass filtered (0.01–0.1 Hz).

### RS FC analysis

Ten large-scale RS networks were created using a seed-region approach [21]. Seed regions for this analysis were all created using the WFU PickAtlas toolbox (http://fmri.wfubmc.edu/software/PickAtlas), part of the SPM12 software (https://www.fil.ion.uclac.uk/spm/software/spm12), merging masks from left and right brain regions (to obtain bilateral masks) included the Automatic Anatomical Labeling (AAL) atlas for the anterior cingulate cortex, precuneus, insula, calcarine cortex, lingual gyrus, fusiform gyrus and cerebellum, and masks derived from the Brodmann atlas for Brodmann Areas 9, 4, 41 and 42. The large-scale RS networks included the anterior and posterior default mode network [22, 23] (DMN I and II; seed regions: bilateral anterior cingulate cortex and precuneus, respectively), executive control network [24] (ECN; seed region: bilateral Brodmann Area 9), salience network [24] (SN; seed region: bilateral insula), primary sensorimotor networks [23] (SMN; seed region: bilateral Brodmann Area 4), primary visual network [23] (seed region: bilateral calcarine cortex), secondary visual networks [23] (secondary visual network I and II; seed regions: bilateral fusiform gyrus and lingual gyrus, respectively), auditory network [23] (seed region: bilateral Brodmann Areas 41&42) and cerebellar network [23] (seed region: bilateral Crus I&II). In addition to large-scale network analysis, a RS FC analysis focused on brain networks having a well-recognized role in the pathophysiology of migraine was done. These included hypothalamic, thalamic, pontine, spinal trigeminal nucleus (STN), periaqueductal gray (PAG) and rostro ventrolateral medulla (RVLM) regions [10]. Region masks for these brain areas were also created using the SPM12 WFU Pickatlas tool. Masks were derived from the AAL atlas for the left and right thalamus. The remaining region masks were created as spheres having a radius of 6 mm, which were centered in the following MNI space coordinates: ± 6, − 6, 10 (for the left and right hypothalamus) [25]; ± 5, − 27, − 27 (for the left and right pons) [26]; ± 4, − 36, − 45 (for the left and right STN) [27]; ± 6, − 30, − 9 (for the left and right PAG) [28]; and ± 6, − 30, − 45 (for the left and right RVLM) [28].

For all networks, Z-score maps of RS FC with each seed region were obtained using the REST software (https://resting-fmri.sourceforge.net) and calculating the correlation coefficients between the time series extracted from each seed region and any other voxel in the brain, followed by the application of a Fisher’s Z transform to improve the gaussianity of the obtained correlation coefficients.

### Sample size estimation

At the time of the study design, no data were available to estimate the effect size for the primary endpoints of the study. Moreover, there were no data regarding a possible carryover effect and its duration upon discontinuation of erenumab (or placebo) on RS FC changes after 3 months of treatment. The sample size calculation for this study was therefore based on the conservative assumption that a relevant carry-over effect was present. As described in literature [29], due to the two-stage nature of fMRI group analysis (i.e., averaging time points within a scan for each patient followed by statistical tests on these averages across patients) the variability (σ) of the effect size consists of two components: (a) A within-scan (i.e., intrasubject) variability (σW) consisting of noise that occurs from one time point to another due to physiological fluctuations, thermal noise and other random factors; (b) A between-patient (i.e., inter-subject) variability (σB), which is the patient-to-patient variability in the effectiveness of the experimental condition in producing a signal change. Estimation of the effect size therefore requires estimating the mean difference (μD) and the variability in its two components (σW and σB).

Based on these scenarios, the sample size of 100 evaluable patients, 50 per treatment group, was chosen as the one which allowed solid results in each subgroup analysis. Considering observing at least a 15% of non-evaluable patients (i.e., fMRI not evaluable or dropout patient), 120 randomized are needed to obtain 100 evaluable patients. The target sample size for this study was therefore 120 patients randomized. In order to obtain 120 randomized patients, about 140 patients were to be screened.

However, due to the COVID-19 pandemic, the study was interrupted balancing the number of available enrolled patients. In fact, the number of 60 randomized patients, 30 per group, guarantees a sufficient level of precision. In detail, considering the evaluation of the difference between treatment groups, about 25 patients are needed to detect a difference between treatment groups of 0.5%, with an intrasubject variability σW of 0.75%, a between-patient variability σB of 0.5%, a power of 90% and an alpha level of 0.002.

With regards to the evaluation of whether the changes of the endpoint are different between the two groups of clinical response within treatment groups: (a) in the erenumab group, considering a percentage of clinical responders of about 30% in migraine patients with previous treatment failure [4], the comparison was between a group of 9 patients (responders) and a group of 21 (non-responders); with this size it is possible to observe a difference (μD) of 0.75%, with an intrasubject variability σW of 0.75%, a between-patient variability σB of 0.5%, a power of 95% and an alpha level of 0.05; (b) in the placebo group, considering a percentage of clinical responders of 15% [4], the comparison was between a group of 5 patients (responders) and a group of 25 (non-responders); with 5 patients it is possible to observe a difference (μD) of 0.75%, with an intrasubject variability σW of 0.75%, a between-patient variability σB of 0.5%, a power of about 80% and an alpha level of 0.05.

### Statistical analysis

The presence of a carry-over effect of treatment was tested, before proceeding with any analysis, by performing two-sample t tests on clinical variables and RS FC maps from all study subjects. Since the results of these analyses suggested the presence of a carry-over effect in the ereneumab/placebo sequence (supplementary results), all analyses were limited to data collected during the first 12-week treatment period, using a parallel group-like design.

Between-group differences in clinical changes from baseline to week 12 were assessed using the Chi-square or Fisher exact test for categorical variables and ANCOVA models, including baseline value and treatment as covariates, for continuous variables (SAS, version 9.4). The difference in least square mean values was extracted as the measure of treatment effect along with corresponding two-sided 95% Confidence Interval (CI).

A propensity score to be used as an independent covariate for statistical analysis of RS FC was created by entering in a logistic regression age, sex, migraine frequency in the 3 months preceding study entry, disease duration and number of previous preventives tried. Average maps of positive RS FC were obtained using scanner-adjusted one-sample t tests, including all time points from all study subjects. Longitudinal RS FC changes within study groups and comparisons of RS FC changes between erenumab and placebo, as well as between responders and non-responders were obtained using propensity score- and scanner-adjusted full factorial models. Results were masked with average maps of positive RS FC within each network. Correlation analyses of longitudinal RS FC changes with patients’ clinical response were run in all migraine patients, and in erenumab and placebo patients, separately, using propensity score- and scanner-adjusted multiple regression models. Results were masked with between-group differences of RS FC changes in the erenumab *vs* placebo group. Finally, RS FC changes during erenumab discontinuation were obtained using propensity score- and scanner-adjusted paired t tests. All between-group comparisons were performed using SPM12 software and reported at *p* < 0.05, family-wise error [FWE] corrected for multiple comparisons. Results were also corrected for the number of investigated networks using the Bonferroni approach (*p* < 0.002). For the carry-over and correlation analysis, results were reported at *p* < 0.05, family-wise error (FWE) corrected and, for explorative purposes, at *p* < 0.001 uncorrected.

Average baseline RS FC Z-scores within the main effects of interest of each network were extracted using REX (https://web.mit.edu/swg/software.htm). These scores were used to investigate the role of baseline RS FC as predictor of response to treatment using propensity-adjusted binary logistic models.

Primary outcomes were analysed on the full analysis set (FAS), i.e., all patients randomized for study treatment, and on the per-protocol set (PPS), i.e., all patients who completed at least the first 3 months of double-blind treatment with no major protocol violation. Secondary outcomes were analysed only on the PPS population.

The analysis protocol of this study was pre-registered with ClinicalTrials.gov, number NCT03977649.

## Results

**Fig. 2:**
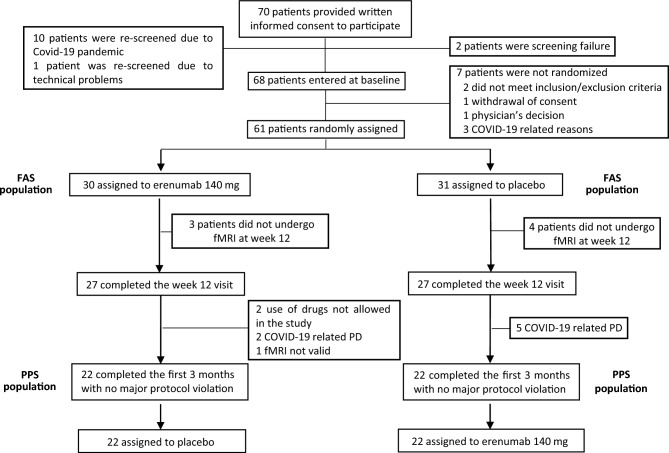
A schematic of patient disposition. *fMRI* functional magnetic resonance imaging, *PD* protocol deviations

Seventy patients were screened for eligibility and 61 were randomized and included in the FAS population. Fifty-four patients, 27 in the erenumab/placebo and 27 in the placebo/erenumab treatment sequence, completed the visit at week 12. Protocol deviations were reported in 10 subjects, related mostly to COVID-19 pandemic. Forty-four enrolled patients, 22 in each treatment sequence, were finally included in the PPS population. Patient disposition is reported in Fig. 2 and supplementary results.

Demographic and clinical characteristics of FAS population at screening are shown in Table 1. No differences were observed between treatment groups**.** Compared to placebo, erenumab patients showed a greater reduction in MMD, MHD, MAT, attack duration, number of days with photophobia, days with phonophobia, ASC-12 and HIT-6 scores from baseline to week 12 (Supplementary Table 1). From week 12 to week 24, during treatment with placebo, these clinical measures increased. Fifteen (56%) erenumab patients and six (22%) placebo patients achieved a 50% reduction in MMD at week 12 vs baseline (*p* = 0·008). Similar results were obtained when analyzing the PPS population (Supplementary results).

**Table 1:** Demographic and clinical characteristics of patients enrolled in the full analysis set (FAS) population at screening

	Treatment sequence AB (erenumab/placebo)	Treatment sequence BA (placebo/erenumab)	Total
Age (years)	48 (30–62)	43 (20–64)	45 (20–64)
Sex (female/male)	27/3	26/5	53/8
Race (Caucasian/Pacific Islander)	29/1	31/0	60/1
Migraine frequency (days/month)	8.9 (4–12)	8.7 (4–12)	8.8 (4–12)
Headache frequency (days/month)	8.9 (4–12)	9.1 (4–14)	9.1 (4–14)
Age at migraine onset	19.1 (5–39)	16.2 (4–43)	17.6 (4–43)
Type of migraine (Aura/No aura/Missing)	1/28/1	3/26/2	4/54/3
Number of patients with any failed prior preventives			
Amitriptyline	21	24	45
Botulinum toxin	4	3	7
Flunarizine	12	11	23
Metoprolol	1	0	1
Pizotifen	3	4	7
Propranolol	14	9	23
Topiramate	14	17	31
Other medications	15	16	31
Number of failed prior medications per patient	3.2 (2–7)	3.3 (2–6)	3.2 (2–7)

Measures are reported as means and ranges. Sex, race, type of migraine and any failed prior preventives are reported as frequencies

Migraine frequency is referring to the monthly number of migraine days defined as any calendar day in which the patient experienced a headache meeting at least one of the following criteria: (1) lasting for ≥ 30 min and meeting one of the following: (a) at least two of the following pain features: unilateral, throbbing, moderate (Numerical Rating Scale: 4–6) to severe (Numerical Rating Scale: 7–10), exacerbated with exercise/physical activity; (b) at least one associated symptom (nausea, vomiting, photophobia, phonophobia); (2) with aura; (3) treated with migraine-specific medication (triptans, ergot derivatives). If these criteria are not fulfilled the calendar day is classified as a headache day. Migraine and headache frequency are the number of days with migraine or headache as recorded in the patients’ diary divided by the number of days of observation multiplied by 30

*MAT* monthly number of days with use of acute treatments, *MHD* monthly headache days, *MMD* monthly migraine days

### RS FC analysis

Average maps of positive RS FC for all considered networks are shown in Supplementary Fig. 1. Regions showing significant RS FC changes, from baseline to week 12 of treatment, within the erenumab and placebo group in the FAS population are described in supplementary results.

#### Between-group RS FC comparison: erenumab vs placebo

**Fig. 3:**
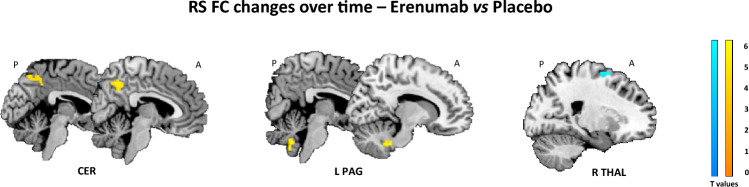
Significant differences in changes from baseline to week 12 of resting state (RS) functional connectivity (FC) in the erenumab vs placebo groups in the full analysis set (FAS) population (propensity score- and scanner-adjusted full factorial models, *p* < 0.05, family-wise error [FWE] corrected for multiple comparisons). Increased RS FC in the erenumab *vs* placebo group is color coded in orange-yellow, while decreased RS FC in the erenumab vs placebo group is color-coded in blue-light. Images are in neurological convention. *A* anterior, *CER* cerebellar network, *P* posterior, *PAG* periaqueductal gray, *THAL* thalamus

Results of the between-group comparison of RS FC changes over time in the erenumab *vs* placebo group are reported in Fig. 3 and Table 2. From baseline to week 12, compared to the placebo group, patients treated with erenumab showed increased RS FC of bilateral precuneus in the cerebellar network and an increased RS FC between the left PAG and cerebellar regions. Compared to patients treated with placebo, migraine patients treated with erenumab also showed decreased RS FC between the right thalamus and the right superior frontal gyrus.

**Table 2:** Regions showing significant differences of resting state (RS) functional connectivity (FC) changes from baseline to week 12 of treatment between erenumab and placebo groups, assessed in the full analysis set (FAS) population (SPM12 full factorial models adjusted for acquisition scanner and propensity score, *p* < 0.05, family-wise error [FWE] corrected for multiple comparisons)

RS networks	Finding	Region	BA	t values	K_E_	MNI coordinates (x y z)
Main large scale RS FC networks						
Cerebellar	Erenumab > Placebo	R precuneus L precuneus	7	4.71* 3.84*	299	2 − 50 46 − 4 − 64 50
RS FC networks relevant for migraine						
L PAG	Erenumab > Placebo	Cerebellum vermis R cerebellum lob IX	–	4.37 4.16	178	6 − 58 − 38 10 − 48 − 40
R thalamus	Placebo > Erenumab	R SFG	6	4.02	110	22 6 64

Results correct for the number of investigated networks using the Bonferroni approach are marked with *

*BA* Brodmann area, *K*_*E*_ cluster extent, *L* left, *R* right, *PAG* periaqueductal gray, *SFG* superior frontal gyrus

#### Between-group RS FC comparison: responders vs non-responders

**Fig. 4:**
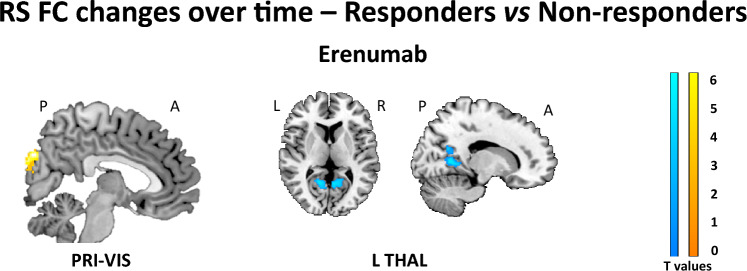
Significant differences in changes from baseline to week 12 of resting state (RS) functional connectivity (FC) in responders vs non-responders in the erenumab group in the full analysis set (FAS) population (propensity score- and scanner-adjusted full factorial models, *p* < 0.05, family-wise error [FWE] corrected for multiple comparisons). Increased RS FC in responders vs non-responders is color coded in orange-yellow, while decreased RS FC in responders vs non-responders is color-coded in blue-lightblue. Images are in neurological convention. *L* left, *R* right, *A* anterior, *P* posterior, *PRI-VIS* primary visual network, *THAL* thalamus

Results of the between-group comparison of RS FC changes over time in responders *vs* non-responders, for the erenumab group are reported in Fig. 4 and Table 3. From baseline to week 12, within the erenumab group, compared to non-responders, responders showed an increased RS FC of the right cuneus in the primary visual network, as well as a decreased RS FC between the left thalamus and bilateral lingual gyrus. No differences of longitudinal RS FC changes were found between responders and non-responders within the placebo group.

**Table 3:** Regions showing significant differences of resting state (RS) functional connectivity (FC) changes from baseline to week 12 between responders and non-responders, within the erenumab group, assessed in the full analysis set (FAS) population (SPM12 full factorial models adjusted for acquisition scanner and propensity score,* p* < 0.05, family-wise error [FWE] corrected for multiple comparisons)

Erenumab group						
RS networks	Finding	Region	BA	t values	K_E_	MNI coordinates (x y z)
Main large scale RS FC networks						
Primary visual	Responders > Non-responders	R cuneus	18	5.59*	188	4 − 84 32
RS FC networks relevant for migraine						
L thalamus	Non-responders > Responders	R lingual gyrus	17	6.74	153	14 − 56 6
		L lingual gyrus	27	5.82*	205	− 8 − 40 2

Results correct for the number of investigated networks using the Bonferroni approach are marked with *

*BA* Brodmann area, *K*_*E*_ cluster extent, *L* left, *R* right

#### Correlation analysis

In the entire group of patients, increased RS FC of the right calcarine cortex within the primary visual network correlated with reduction in MMD at week 12. Significant correlations were also found between decreased right thalamic RS FC with the left SFG and reduction in MMD and ASC-12 scores, decreased right thalamic RS FC with the right SFG and reduction in HIT-6 scores, and between the decreased right thalamic RS FC with the left SFG and middle cingulate cortex and reduction in the number of days with phonophobia. Increased RS FC of the left precuneus within the cerebellar network correlated with the reduction in the number of days with photophobia at week 12. No significant correlations were found in the erenumab and placebo group separately (Supplementary Table 6).

#### Prediction analysis

A lower baseline Z-score in the right pontine network was associated with higher odds of a good clinical response to erenumab after 12 weeks of treatment (odds ratio = 0·95, 95% CI = 0·92–0·99, *p* = 0·03). This pontine network comprised the functional interaction between the right pons and bilateral cerebellum, occipital, frontal, parietal and temporal brain areas. The remaining baseline Z-scores were not significantly associated with 12-week clinical response.

#### RS FC changes during erenumab discontinuation

During erenumab discontinuation, patients showed increased RS FC of the left superior frontal gyrus within the primary visual network. Moreover, patients showed decreased RS FC between the left PAG and left SMA, between bilateral PAG and left cerebellum, and between the right pons and left superior temporal gyrus (Supplementary Table 7).

#### Safety

No safety concerns emerged during the study. One serious AE was reported in a patient receiving erenumab (femur fracture), and treatment emergent AEs were essentially limited to constipation (10%) and upper respiratory tract infections (7%). Treatment emergent adverse events are described in Supplementary Table 13. No significant changes in laboratory parameters or physical findings were observed.

## Discussion

This study confirmed the efficacy of a 12-week erenumab treatment in episodic migraine and showed that the treatment is associated to RS FC changes within clinically relevant brain networks mediating migraine manifestations. In line with previous clinical studies [30], we showed for the first time that erenumab-related brain functional changes are temporary and tend to reverse when erenumab is stopped. In accordance with previous findings, we found a placebo response rate of 22% and we confirmed placebo-related functional changes in nociceptive brain networks that may influence pain perception [31]. fMRI studies showed that placebo treatment could modulate the activity of opioidergic and dopaminergic networks including brain areas that are part of the descending pain inhibitory system, such as the hypothalamus, PAG, anterior cingulate and dorsolateral prefrontal cortex, thus reducing the pain perception [31].

Compared to placebo, patients receiving erenumab showed increased cerebellar RS FC with the precuneus, an area implicated in sensory integration and mind wandering from pain [32], and the PAG, a pivotal region of the pain inhibitory system [33]. Interestingly, the higher RS FC between the cerebellum and precuneus was associated with improvement of photophobia. The cerebellum is functionally and structurally connected with cortical and subcortical areas implicated in multisensory processing and in the modulation of the affective component of pain [34]. Moreover, our findings corroborate previous evidence showing cerebellar alterations in migraine patients, further supporting its involvement in migraine [25, 35].

We also found a reduced thalamic RS FC with frontal brain areas involved in nociception in migraine patients treated with erenumab. The thalamus is a central area for pain modulation, where peripheral nociceptive trigeminal inputs converge before reaching the cortex [36]. Thalamocortical projections to sensory-motor, visual, auditory and limbic areas are modulated by different pathways involved in emotion, cognition and autonomic responses [37]. Numerous neuroimaging studies demonstrated structural and functional thalamic alterations in migraine patients during [38, 39] and outside [39, 40] the migraine attack, which could explain part of the complexity of migraine features. An abnormal thalamic activation may account for the development of sensory hypersensitivity and cutaneous allodynia in migraineurs [41, 42]. Of note, we showed a significant association between decreased thalamic RS FC and reduction in attack frequency, migraine impact on daily life and migraine severity, in terms of reduction of cutaneous allodynia and phonophobia. Moreover, a higher reduction of thalamic RS FC was found in patients who responded to erenumab compared to those who did not respond. Based on these findings, it is tempting to speculate an association between thalamic activity and therapeutic effect of erenumab. Our hypothesis is in line with previous preclinical and clinical studies suggesting that the benefit of gepants and acute migraine-specific therapies, such as lasmiditan and triptans, in stopping the acute migraine attack could involve the modulation of thalamic neurons [36, 43, 44].

Clinical efficacy of erenumab was also linked to functional modulation of primary and secondary visual areas. We found that responders to erenumab experienced a higher RS FC between the calcarine cortex and extrastriate visual areas, like the cuneus, compared to non-responders. The role of the visual network in migraine, regardless the presence of aura, is well established [10]. Similarly to our results, previous imaging studies demonstrated a significant association between functional and structural abnormalities of visual areas and migraine attack frequency and severity [25, 45], corroborating the presence of a strict interplay between the visual, thalamic and trigeminal networks [45–47].

Only one previous observational study [12] has explored central effects of 2-week therapy of erenumab 70 mg demonstrating a decreased activation of the thalamus, cerebellum and nociceptive cortical areas in response to trigeminal stimulation, as well as reduced hypothalamic activation in patients who reported a 30% decrease of MHD. A more pronounced reduction of the hypothalamic activity during trigeminal stimulation has also been found in migraineurs who had a 30% reduction of MHD after three months of galcanezumab [11]. Following the International Headache Society guidelines for RCTs for prevention of episodic migraine [18], we considered responders those patients achieving at least a 50% reduction in MMDs. The same definition of patients’ responders was used in a recent observational study that have examined changes in RS FC and fMRI activation after extracranial thermal pain between erenumab responders and non-responders after 8 weeks of treatment. This study showed greater pain-induced activity of the PAG, frontal and cingulate cortex in erenumab responders compared to non-responders, as well as increased RS FC of the hypothalamus, temporal, parietal and frontal brain areas in patients who responded to erenumab. Differences in the fMRI approaches, the statistical thresholds applied, in the clinical characteristic of patients and treatment duration might explain the discrepancy between ours and previous findings.

Brain functional modifications observed after 12 weeks of erenumab at the level of PAG reversed when treatment was stopped. This finding is in line with clinical data [30] showing a loss of therapeutic effects and progressive clinical worsening of migraineurs during discontinuation of anti-CGRP mAbs. We cannot exclude that longer treatment may be associated with stability of the changes.

The identification of patients who might benefit more of a given treatment is an important goal of the expanding scenario of migraine treatment. In this perspective, we found that a lower RS FC of the pontine network contributes to identify patients with a higher probability of responding to erenumab. The pontine area is one of the key players of migraine pathophysiology. Several pieces of evidence [10] suggested that the pons, along with the hypothalamus, could be putative drivers of the acute migraine attack. A recent observational study showed that an altered activity of the STN, a brainstem area functionally connected to the pons that plays a crucial role in migraine pain [37], could predict a better response to galcanezumab, a mAb targeting the CGRP ligand [11]. Taken together, these findings indicate that brainstem activity could be an imaging biomarker that predicts success of mAbs targeting the CGRP pathway in migraine prevention. This possible treatment response biomarker should be validated in larger future studies.

The mechanisms underlying the therapeutic benefit of anti-CGRP mAbs in migraine prevention are a matter of ongoing discussion. Given the large size of mAbs targeting the CGRP pathway, the most promoted idea is that their site of action is outside the BBB and may include meningeal receptors, trigeminal sensory fibers and the trigeminal ganglion [2]. Our results point to central effects strictly related to the period of erenumab administration, which could be an indirect effect of meningeal receptors or trigeminal ganglion modulation. On the other hand, the improvement of migraine symptoms that are purely centrally mediated, such as phothophobia and phonophobia could lead to speculation that the small amount of mAbs penetrating the BBB or entering the CSF through the choroid plexus could exert an effect at the level of the central nervous system. Moreover, brain changes detected after 12 weeks of erenumab involved those brain areas, including the thalamus, brainstem and cerebellum, where the CGRP receptor is densely expressed [36, 48]. However, we cannot exclude that the functional changes we have observed in brain areas involved in centrally mediated migraine symptoms could be a consequence of the improvement of such symptomatology.

To our knowledge, this is the first study investigating erenumab central effects after 12 weeks of treatment using a randomized, double-blind, placebo-controlled design. Another strength of this study is the inclusion of only episodic migraine patients, who were not taking other migraine preventives and who were studied outside their migraine attack. In addition, we have applied a statistical approach corrected for multiple comparisons, thus limiting the number of false positives.

We are aware that a limitation of this study is the lack of a healthy control group to serve as reference for RS FC findings. Information regarding the time elapsed between the MRI and the following migraine attack was also missing. Moreover, the sample size of the migraine subgroups was quite small, increasing the risk of false positive results, and our migraine sample was heterogeneous, including both patients with and without aura. Lastly, the software we used for our voxel-wise comparisons (i.e., SPM12) does not include a full implementation of mixed models. Larger studies are needed to confirm our findings. Although a peripheral mechanism of action of erenumab seems the most likely, this study suggests that clinical benefit of erenumab could derive from combined peripheral and central mechanisms.

### Supplementary Information

Below is the link to the electronic supplementary material.

Supplementary file1 (DOCX 982 KB)

## Data Availability

Data supporting the findings of this study are available from the corresponding author, upon reasonable request.
