# Supplementary material for: The effect of erenumab on brain network function in episodic migraine patients: a randomized, placebo-controlled clinical trial (RESET BRAIN)
Source: J Neurol. 2023 Aug 8;270(11):5600–12. doi: 10.1007/s00415-023-11879-9 (PMC10576673; doi:10.1007/s00415-023-11879-9)
Supplement: Supplementary file 1 — Supplementary file1 (DOCX 982 KB) [file 415_2023_11879_MOESM1_ESM.docx]

**Supplementary Methods**

**Inclusion and exclusion criteria**

Inclusion criteria

1. Adult patients, aged between 18 and 65 years;

2. History of migraine with or without aura for at least 12 months prior to screening according to International Classification of Headache Disorders (ICHD-3), based on medical records and/or self-reported by patients;

3. Migraine frequency: ≥ 4 and < 15 migraine days per month on average across the 3 months prior to screening and confirmed during the run-in phase based on headache diary calculation;

4. Headache frequency: <15 headache days per month on average across the 3 months prior to screening and confirmed during the run-in phase based on headache diary calculation;

5. Failure to two or more previous treatment categories locally indicated for migraine prophylaxis due to either lack of efficacy or poor tolerability.

Efficacy failure was defined as "no reduction in headache frequency, duration, or severity after administration of the medication for at least 6 weeks at approved therapeutic dose(s) based on the investigator's assessment at any time prior to screening." Tolerability failure was defined as "documented discontinuation of the medication due to adverse events at any time prior to screening".

Exclusion criteria

1. Older than 50 years of age at migraine onset;

2. History of cluster headache or hemiplegic migraine headache;

3. Unable to differentiate migraine from other headaches;

4. History of chronic pain disorders and neuropathic pain;

5. History of head trauma or seizure or major psychiatric disorders or suicidal ideation/behavior at any time before screening;

6. Currently receiving any other prophylactic treatment for migraine and/or prohibited medications, non-pharmacologic interventions or devices (any substance, non-pharmacologic intervention or device acting on the central nervous system), or less than 60 days or 5 half-lives prior to the start of the run-in period, during the run-in period, or treatment period;

7. Exposure to botulinum toxin in the head and/or neck region within 4 months prior to the start of the run-in period, during the run-in period, or treatment period;

8. Exposure to the following for any indication in any month during the 2 months prior to the start of the run-in period: ergotamines or triptans for ≥ 10 days per month, or simple analgesics (non-steroidal anti-inflammatory drugs [NSAIDs], acetaminophen) for ≥ 15 days per month, or opioid- or butalbital-containing analgesics for ≥ 4 days per month;

9. History of hypersensitivity to any of the study treatments or their excipients or to drugs of similar chemical classes;

10. Use of other investigational drugs within 5 half-lives of initiation of study treatment or within 30 days (e.g., small molecules) or until the expected pharmacodynamic effect had returned to baseline (e.g., biologics), whichever is longer; or longer if required by local regulations;

11. Previous exposure to erenumab or exposure to any other prophylactic CGRP-targeted therapy (prior to and during the study);

12. History of human immunodeficiency virus (HIV) infection;

13. History of malignancy of any organ system (other than localized basal cell carcinoma of the skin or in situ cervical cancer), treated or untreated, within the past 5 years, regardless of whether there was evidence of local recurrence or metastases;

14. History or evidence of any other unstable or clinically significant medical condition that in the opinion of the investigator would have posed a risk to subject safety or interfered with the study evaluation, procedures, or completion;

15. Any clinically significant vital sign, laboratory, or electrocardiogram (ECG) abnormality during screening that, in the opinion of the investigator, could have posed a risk to subject safety or interfered with the study evaluation;

16. Myocardial infarction, stroke, transient ischemic attack, unstable angina, or coronary artery bypass surgery or other re-vascularization procedures within 6 months prior to screening;

17. Evidence of drug or alcohol abuse or dependence within 12 months prior to screening based on medical records or self-reported by the patient;

18. Pregnant or breastfeeding;

19. All contraindications for MRI scan, including but not limited to presence of pacemakers not compatible with MRI, aneurysm clips, artificial heart valves, ear implants, or foreign metal objects in the eyes, skin, or body that would have contraindicated an MRI scan or any other clinical history or examination finding that, in the judgment of the investigator, would have posed a potential hazard in combination with MRI.

**Definition of migraine day and duration of a migraine attack**

A migraine day was defined as any calendar day in which the patient experienced a qualified migraine headache, defined as a headache meeting at least one of the following criteria:

1) Lasting for ≥ 30 minutes and meeting one of the following:

a) At least two of the following pain features: unilateral, throbbing, moderate (Numerical Rating Scale: 4-6) to severe (Numerical Rating Scale: 7-10), exacerbated with exercise/physical activity;

b) At least one associated symptom (nausea, vomiting, photophobia, phonophobia);

2) With aura;

3) Treated with migraine-specific medication (triptans, ergot derivatives).

Monthly migraine days were the number of days with migraine as recorded in the patients’ diary divided by the number of days of observation multiplied by 30.

If less than 48 hours of freedom from headache occurred between two migraine attacks, they were considered as part of the same attack and the duration of migraine attack was calculated as the sum of durations of each single period. Otherwise, they were considered as distinct attacks.

**MRI acquisition procedures**

The following 3.0 T scanners were used at each site: IRCCS San Raffaele Scientific Institute, Milan: Philips Ingenia; Istituto Neurologico Mondino, Pavia: Siemens Skyra; Istituto Neurologico Besta, Milan: Philips Achieva; University of Campania ‘Luigi Vanvitelli’, Naples, and SS Filippo e Nicola Hospital, Avezzano: GE Discovery MR 750. No major hardware/software upgrades occurred during the study. Total acquisition time of the RS fMRI sequence was 8 min 20 s.

According to instructions provided in the MRI manual, axial MRI sequences were always positioned to run parallel to a line that joins the most infero-anterior and infero-posterior parts of the corpus callosum. The same patient positioning procedure was used at each time point to achieve the same slice positioning. Before centralized MRI analysis, a quality check (QC) was performed on scans at the Neuroimaging Research Unit, IRCCS San Raffaele Scientific Institute, Milan. The QC included an inspection of MRI sequence parameters (which should comply with the approved dummy-run), and an evaluation of positioning/repositioning at follow-up and of the presence of artefacts. All acquired scans passed the centralized QC.

**Supplementary Results**

Seventy patients were screened for eligibility and 61 were randomized and included in the FAS population, 30 in the erenumab/placebo sequence and 31 in the placebo/erenumab sequence. Two patients were screening failure and did not enter the run-in phase, three patients were not randomized due to COVID-19 related reasons, two did not meet inclusion/exclusion criteria, one chose to withdraw from the study and one was withdrawn following the physician’s decision. Fifty-four patients, 27 in the erenumab/placebo and 27 in the placebo/erenumab treatment sequence, completed the fMRI visit at week 12. Forty-four enrolled patients, 22 in each treatment sequence, were finally included in the PPS population. In the erenumab/placebo sequence, two patients were excluded from the PPS population due to the use of drugs not allowed in the study and one patient was exclude to due invalid fMRI exam. The remaining patients in the erenumab/placebo and placebo/erenumab sequence were excluded from the PPS population to due protocol deviations related mostly to assessments not being performed caused by the COVID-19 pandemic.

**Carry-over effect.** A carry-over effect for the erenumab/placebo sequence was demonstrated on clinical measures and in all large-scale RS networks, except for the SN, as well as in the left hypothalamic, bilateral thalamic and left RVLM network (Supplementary Table 2 and 3). We found that the effect of erenumab on MMDs at week 16 was still present in patients randomized to treatment sequence erenumba/placebo. (Supplementary Table 3).

**Within-group RS FC changes over time in the FAS population.** RS FC changes detected at week 12 *vs* baseline in the erenumab group are reported in supplementary Figure 2A and supplementary Table 4. Compared to baseline, erenumab patients showed increased RS FC between the left PAG and ipsilateral supplementary motor area (SMA), as well as between cerebellar regions, the right PAG and bilateral pons.

RS FC changes detected at week 12 *vs* baseline in the placebo group are reported in supplementary Figure 2B and supplementary Table 5. Compared to baseline, at week 12 placebo patients showed RS FC decrease in frontal, parietal, occipital and temporal regions of the DMN, ECN, SN and visual networks. Decreased RS FC between the left PAG and right cerebellum, of the bilateral thalamus with precuneus, angular gyrus and calcarine cortex, and of the right hypothalamus with the left cuneus was also detected.

**Results in PPS population.** The PPS population showed similar demographic and clinical data to the FAS population (supplementary Table 8). Similarly to the FAS population, in the PPS population, patients treated with erenumab showed a greater reduction in MMD, MHD, MAT, duration of attacks, number of days with photophobia, days with phonophobia, NRS, ASC-12 and HIT-6 and HADS-A scores from baseline to week 12, compared to placebo (supplementary Table 9).

Within-group RS FC changes over time. At week 12 *vs* baseline, erenumab patients showed increased RS FC between the left and right PAG and left cerebellum and SMA (supplementary Table 10 and supplementary Figure 3A). Compared to baseline, at week 12 placebo patients showed RS FC decrease in parietal, occipital, temporal and cerebellar regions of the SN, auditory and visual networks. Placebo patients experienced also decreased RS FC between the left PAG and right cerebellum, of the bilateral thalamus with precuneus, and of the right hypothalamus with the left cuneus and precuneus. Finally, placebo patients showed increased RS FC in parietal regions of the auditory network, as well as between the right thalamus and bilateral SMA (supplementary Table 11 and supplementary Figure 3B).

Between-group RS FC comparison: erenumab *vs* placebo. From baseline to week 12 of treatment, compared to placebo, erenumab patients showed increased RS FC of bilateral precuneus in the cerebellar network, and of right calcarine cortex in the primary visual network (supplementary Table 12 and supplementary Figure 4A). Compared to placebo, erenumab patients showed also an increased RS FC between cerebellar regions and the left PAG, as well as decreased RS FC between the right thalamus and right superior frontal gyrus (supplementary Table 12 and supplementary Figure 4B).

Between-group RS FC comparison: responders *vs* non-responders. No differences of longitudinal RS FC changes were found between responders and non-responders within the erenumab and placebo group.

**Supplementary Table 1.** Clinical characteristics over the treatment period in the full analysis set (FAS) population

|  | **Treatment sequence Erenumab/Placebo** | | | **Treatment sequence Placebo/Erenumab** | | | **Between-treatment difference**  **(Baseline vs week 12)** |
| --- | --- | --- | --- | --- | --- | --- | --- |
|  | **Baseline** | **Week 12** | **Week 24** | **Baseline** | **Week 12** | **Week 24** | **Difference of Least Square Means**  **(95% C.I.), *p*-value** |
| **MMD**  *Change from baseline* | 9.02 (2.62)  - | 4.14 (2.55)  -4.93 | 8.26 (3.48)  -0.81 | 9.37 (2.78)  - | 8.10 (4.40)  -1.12 | 3.61 (2.26)  -5.76 | -3.918 (-5.880, -1.956)  0.0002 |
| **Duration of migraine attacks**  *Change from baseline* | 16.99 (10.03)  - | 9.06 (5.66)  -8.51 | 6.42 (13.48)  -1.15 | 13.90 (9.27)  - | 13.48 (11.01)  -0.74 | 6.96 (5.09)  -7.63 | -5.64 (-10.16, -1.12)  0.0155 |
| **MHD**  *Change from baseline* | 9.87 (2.77)  - | 4.80 (2.75)  -5.09 | 8.66 (4.09)  -1.24 | 9.68 /2.96)  - | 8.21 (4.40)  -1.32 | 3.91 (2.41)  -5.80 | -3.53 (-5.52, -1.55)  0.0008 |
| **MAT**  *Change from baseline* | 7.76 (2.97)  - | 3.59 (2.54)  -4.14 | 7.44 (3.17)  -0.29 | 7.88 (3.00)  - | 7.73 (4.32)  0.14 | 3.21 (2.19)  -4.55 | -4.18 (-6.09, -2.26)  <0.0001 |
| **Number of days with photophobia**  *Change from baseline* | 5.17 (3.53)  - | 1.79 (2.01)  -2.99 | 4.04 (2.89)  -0.74 | 5.90 (3.80)  - | 4.79 (4.25)  -0.96 | 2.20 (2.15)  -3.74 | -2.69 (-4.46, -0.93)  0.003 |
| **Number of days with phonophobia**  *Change from baseline* | 4.67 (3.88)  - | 1.12 (1.83)  -3.44 | 4.20 (2.62)  -0.35 | 6.47 (3.38)  - | 5.24 (4.43)  -0.86 | 2.31 (2.12)  -3.89 | -3.48 (-5.22, -1.74)  0.0002 |
| **Number of days with nausea**  *Change from baseline* | 4.93 (3.15)  - | 2.26 (2.28)  -2.62 | 5.55 (3.86)  0.67 | 3.90 (3.45)  - | 2.92 (3.12)  -1.43 | 1.23 (1.65)  -2.79 | -0.819 (-2.263, 0.625)  0.26 |
| **Number of days with aura**  *Change from baseline* | 0.50 (1.33)  - | 0.22 (0.60)  -0.32 | 0.48 (1.09)  -0.06 | 0.68 (1.32)  - | 0.04 (0.23)  -0.64 | 0.07 (0.25)  -0.68 | 0.19 (-0.04, 0.43)  0.10 |
| **NRS scores**  *Change from baseline* | 6.61 (1.04)  - | 5.95 (1.96)  -0.60 | 6.67 (1.21)  0.12 | 6.70 (1.07)  - | 6.84 (1.69)  -0.01 | 5.89 (2.10)  -0.74 | -0.59 (-1.43, 0.25)  0.17 |
| **HIT-6 scores**  *Change from baseline* | 64.73 (3.88)  - | 53.35 (9.96)  -11.69 | 63.23 (5.82)  -1.81 | 66.06 (5.05)  - | 61.33 (6.49)  -4.89 | 57.41 (7.82)  -8.26 | -7.06 (-11.29, -2.82)  0.0016 |
| **ASC-12 scores**  *Change from baseline* | 6.13 (4.89)  - | 3.58 (3.88)  -2.85 | 5.81 (5.44)  -0.62 | 5.45 (4.22)  - | 5.52 (4.08)  0.15 | 4.64 (4.75)  -0.96 | -2.46 (-4.30, -0.61)  0.0102 |
| **HAD-A scores**  *Change from baseline* | 6.60 (3.97)  - | 3.85 (3.09)  -2.50 | 4.81 (3.70)  -1.54 | 5.42 (3.74)  - | 4.67 (2.84)  -0.85 | 4.00 (3.65)  -1.22 | -1.22 (-2.49, 0.04)  0.057 |
| **HAD-D scores**  *Change from baseline* | 5.60 (3.23)  - | 3.12 (3.00)  -2.19 | 5.12 (3.55)  -0.19 | 5.29 (3.90)  - | 4.19 (2.99)  -1.30 | 3.93 (4.16)  -1.33 | -0.99 (-2.38, 0.39)  0.16 |

Duration of migraine attacks was considered in hours.

Abbreviations: Allodynia Symptom Checklist (ASC-12); Confidence Interval (C.I.); Hospital Anxiety and Depression Scales (HAD-A and HAD-D); Headache Impact Test-6 (HIT-6); Monthly number of days with use of Acute Treatments (MAT); Monthly Headache Days (MHD); Monthly Migraine Days (MMD); Numerical Rating Scale (NRS).

**Supplementary Table 2.** Regions showing a carry over effect, i.e., showing significant differences in the sum of changes over time of resting state (RS) functional connectivity (FC) between the two study sequences (i.e., Erenumab/Placebo *vs* Placebo/Erenumab). The analysis was performed using SPM12 two-sample t tests adjusted for acquisition scanner and propensity score (*p*<0.001, uncorrected, cluster extent k=50. Results at *p*<0.05, family-wise error [FWE] corrected for multiple comparisons are marked with *).

| **RS FC networks relevant for migraine** | | | | | | | |
| --- | --- | --- | --- | --- | --- | --- | --- |
| **RS networks** | **Finding** | **Region** | **BA** | **t values** | **K_E_** | **MNI coordinates**  **(x y z)** |  |
| L hypothalamus | Placebo/Erenumab > Erenumab/Placebo | L Amygdala | 25 | 4.84 | 60 | -10 10 -16 |  |
| L Thalamus | Placebo/Erenumab > Erenumab/Placebo | L MTG | 37 | 4.89 | 58 | 60 -58 4 |  |
| L RVLM | Erenumab/Placebo > Placebo/Erenumab | L postcentral gyrus | 4 | 4.28 | 54 | -44 -10 42 |  |
| R Thalamus | Placebo/Erenumab > Erenumab/Placebo | R MTG | 21 | 4.32 | 112 | 62 -54 6 |  |
| DMN I | Erenumab/Placebo > Placebo/Erenumab | R angular gyrus | 7 | 4.55* | 104 | 44 -64 56 |  |
| ECN | Erenumab/Placebo > Placebo/Erenumab | L MFG | 9 | 4.31 | 52 | -30 28 42 |  |
| SMN | Placebo/Erenumab > Erenumab/Placebo | L OFC | 11 | 4.29 | 70 | -4 46 -10 |  |
| Secondary visual I | Placebo/Erenumab > Erenumab/Placebo | L ACC | 24 | 4.26* | 125 | 0 30 -6 |  |
| Secondary visual II | Erenumab/Placebo > Placebo/Erenumab | R SPL | 7 | 4.44 | 62 | 28 -68 50 |  |
| Auditory | Erenumab/Placebo > Placebo/Erenumab | R postcentral gyrus | 3 | 4.28 | 66 | 26 -38 56 |  |
| Cerebellar | Placebo/Erenumab > Erenumab/Placebo | L cerebellum lobule VIII | - | 4.70 | 58 | -14 -62 -34 |  |

Abbreviations: ACC=Anterior cingulate cortex; BA=Brodmann area; DMN=default-mode network; ECN=executive control network; K_E_=cluster extent; L=left; MFG=middle frontal gyrus; MTG=middle temporal gyrus; OFC=orbitofrontal cortex; R=right; RVLM=rostro-ventrolateral medulla; SMN=sensorimotor network; SPL=superior parietal lobule.

**Supplementary Table 3.** Changes of monthly migraine days from baseline to week 24.

| **Treatment sequence AB (erenumab/placebo)** | | |
| --- | --- | --- |
| **Treatment** | **Time point** | **MMDs**  *Change from baseline* |
|  | Baseline | 8.74 (2.67) |
| Erenumab | Week 4 | 4.96 (2.64)  -3.77 |
|  | Week 8 | 4.20 (2.39)  -4.5.4 |
|  | Week 12 | 3.96 (2.52)  -4.77 |
| Palacebo | Week 16 | 5.15 (2.97)  -3.59 |
|  | Week 20 | 6.96 (3.51)  -1.77 |
|  | Week 24 | 8.72 (3.46)  -0.01 |
| **Treatment sequence BA (placebo/erenumab)** | | |
| **Treatment** | **Time point** | **MMDs**  *Change from baseline* |
|  | Baseline | 9.04 (2.73) |
| Placebo | Week 4 | 8.50 (2.96)  -0.54 |
|  | Week 8 | 7.80 (3.50)  -1.24 |
|  | Week 12 | 8.77 (4.38)  -0.27 |
| Erenumab | Week 16 | 5.54 (3.65)  -3.51 |
|  | Week 20 | 4.97 (2.67)  -4.16 |
|  | Week 24 | 3.93 (2.33)  -5.19 |

**Supplementary Table 4.** Regions showing significant resting state (RS) functional connectivity (FC) changes, from baseline to week 12 of treatment, within the erenumab group, assessed in the full analysis set (FAS) population (SPM12 full factorial models adjusted for acquisition scanner and propensity score, *p* <0.05, family-wise error [FWE] corrected for multiple comparisons. Results correct for the number of investigated networks using the Bonferroni approach are marked with *).

| **RS FC networks relevant for migraine** | | | | | | |
| --- | --- | --- | --- | --- | --- | --- |
| **RS networks** | **Finding** | **Region** | **BA** | **t values** | **K_E_** | **MNI coordinates**  **(x y z)** |
| L PAG | Increased RS FC | L SMA | 8 | 5.71 | 90 | -4 22 60 |
| L pons | Increased RS FC | L cerebellum lob VI | - | 5.02* | 194 | -32 -54 -34 |
| R PAG | Increased RS FC | L cerebellum crus I | - | 5.72 | 154 | -28 -60 -36 |
| R pons | Increased RS FC | L cerebellum crus I | - | 5.10 | 113 | -26 -74 -34 |

Abbreviations: BA=Brodmann area; K_E_=cluster extent; L=left; PAG=periaqueductal gray; R=right; SMA=supplementary motor area.

**Supplementary Table 5.** Regions showing significant resting state (RS) functional connectivity (FC) changes, from baseline to week 12 of treatment, within the placebo group, assessed in the full analysis set (FAS) population (SPM12 full factorial models adjusted for acquisition scanner and propensity score, *p* <0.05, family-wise error [FWE] corrected for multiple comparisons. Results correct for the number of investigated networks using the Bonferroni approach are marked with *).

| **Main large scale RS FC networks** | | | | | | |
| --- | --- | --- | --- | --- | --- | --- |
| **RS networks** | **Finding** | **Region** | **BA** | **t values** | **K_E_** | **MNI coordinates**  **(x y z)** |
| DMN II | Decreased RS FC | R SFG | 10 | 6.31 | 95 | 16 60 6 |
|  |  | R MTG | 21 | 5.60 | 140 | 66 -8 -16 |
| ECN | Decreased RS FC | L Precuneus | 7 | 5.24 | 129 | -10 -54 46 |
| Primary visual | Decreased RS FC | L hippocampus | 20 | 5.72 | 114 | -30 -8 -18 |
| Secondary visual II | Decreased RS FC | L STG | 48 | 5.51* | 187 | -48 -14 -6 |
| SN | Decreased RS FC | R lingual gyrus | 17 | 6.31* | 380 | 8 -74 2 |
| **RS FC networks relevant for migraine** | | | | | | |
| L PAG | Decreased RS FC | R cerebellum lob IX | - | 5.40* | 185 | 2 -60 -48 |
| L thalamus | Decreased RS FC | L angular gyrus | 7 | 6.72* | 693 | -38 -72 44 |
|  |  | L precuneus | 31 | 5.24* | 210 | -2 -54 18 |
| R hypothalamus | Decreased RS FC | L cuneus | 17 | 4.68 | 119 | -6 -68 26 |
| R thalamus | Decreased RS FC | L precuneus | 7 | 4.81 | 133 | -10 -50 42 |
|  |  | L calcarine cortex | 17 | 4.72* | 214 | -2 -62 10 |

Abbreviations: BA=Brodmann area; K_E_=cluster extent; DMN=default-mode network; ECN=executive control network; SN=salience network; L=left; PAG=periaqueductal gray; R=right; SFG=superior frontal gyrus; MTG=middle temporal gyrus; STG=superior temporal gyrus.

**Supplementary Table 6.** Correlations of changes in RS FC over time with changes in patients’ clinical response in the per protocol set (PPS) population (SPM12 multiple regression models adjusted for acquisition scanner and propensity score, p<0.001, uncorrected).

| **All patients** | | | | | | |
| --- | --- | --- | --- | --- | --- | --- |
| **Clinical variable** | **RS networks** | **Region** | **BA** | **K_E_** | **MNI coordinates**  **(x y z)** | **r** |
| Monthly migraine days | Primary visual | R calcarine cortex | 17 | 5 | 2 -76 6 | 0·52 |
|  | R thalamus | L SFG | 6 | 5 | -16 -4 66 | -0·53 |
| HIT-6 score | R thalamus | R SFG | 6 | 13 | 24 -2 64 | -0·53 |
| Severity of migraine pain | Primary visual | R calcarine cortex | 17 | 29 | 4 -80 4 | 0·64 |
| ASC-12 score | R thalamus | L SFG | 6 | 5 | -20 -2 62 | -0·52 |
| Number of days with photophobia | Cerebellar | L precuneus | 7 | 5 | -6 -64 50 | 0·57 |
| Number of days with phonophobia | R thalamus | L SFG | 6 | 5 | -18 2 64 | -0·58 |
|  |  | R MCC | 24 | 5 | 0 10 32 | -0·56 |

Abbreviations: ASC-12=allodynia symptom checklist; BA=Brodmann area; K_E_=cluster extent; HIT-6= Headache impact test; L=left; MCC=middle cingulate cortex; R=right; SFG=superior frontal gyrus.

**Supplementary Table 7.** RS FC changes during erenumab discontinuation in the per protocol set (PPS) population (i.e., RS FC changes at week 24 compared to week 12 *vs* those detected at week 12 *vs* baseline, SPM12 paired t test adjusted for acquisition scanner and propensity score, *p* <0.05, family-wise error [FWE] corrected for multiple comparisons. Results correct for the number of investigated networks using the Bonferroni approach are marked with *).

| **Main large scale RS FC networks** | | | | | | |
| --- | --- | --- | --- | --- | --- | --- |
| **RS networks** | **Finding** | **Region** | **BA** | **t values** | **K_E_** | **MNI coordinates**  **(x y z)** |
| Primary visual I | Increased RS FC | L SFG | 32 | 5.16* | 321 | -16 24 48 |
| **RS FC networks relevant for migraine** | | | | | | |
| L PAG | Decreased RS FC | L SMA | 8 | 4.62 | 130 | -6 22 60 |
|  |  | L cerebellum lob VI | - | 5.08 | 94 | -30 -54 -35 |
| R PAG | Decreased RS FC | L cerebellum crus I | - | 6.24* | 227 | -30 -60 -38 |
| R pons | Decreased RS FC | L STG | 48 | 5.02 | 169 | -52 -26 4 |

Abbreviations: BA=Brodmann area; K_E_=cluster extent; PAG=periaqueductal gray; SFG=superior frontal gyrus; SMA=supplementary motor area; STG=superior temporal gyrus.

**Supplementary Table 8.** Demographic and clinical characteristics of patients enrolled in the per protocol set (PPS) population at screening.

|  | **Erenumab/Placebo treatment sequence**  **(N=22)** | **Placebo/Erenumab treatment sequence**  **(N=22)** | **Total**  **(N=44)** |
| --- | --- | --- | --- |
| **Age** (years) | 48·1 (8·83) | 42·6 (11·31) | 45·4 (10·41) |
| **Sex** (female/male) | 19/3 | 20/2 | 39/5 |
| **Race (**Caucasian/Pacific Islander) | 21/1 | 22/0 | 43/1 |
| **Migraine frequency*** (days/month) | 9·1 (1·94) | 9·1 (1·97) | 9·1 (1·93) |
| **Headache frequency*** (days/month) | 9·2 (1·99) | 9·6 (2·26) | 9·4 (2·11) |
| **Age at migraine onset** (years) | 19·2 (9·37) | 16·8 (10·20) | 18·0 (9·75) |
| **Type of migraine** (Aura/No aura/Missing) | 1/20/1 | 1/20/1 | 2/40/2 |
| **Number of patients with any failed prior preventives**  *Amitriptyline*  *Botulinum toxin*  *Flunarizine*  *Metoprolol*  *Pizotifen*  *Propranolol*  *Topiramate*  *Other medications* | 14  4  9  1  2  11  10  12 | 16  2  8  0  4  5  11  12 | 30  6  17  1  6  16  21  24 |
| **Number of failed prior medications per patient** | 3·3 (1·32) | 3·3 (1·32) | 3·3 (1·30) |

* Attack frequency in the 3 months preceding study entry.

**Supplementary Table 9.** Clinical characteristics over the treatment period in the PPS population.

|  | **Erenumab/Placebo treatment sequence** | | | **Placebo/Erenumab treatment sequence** | | | **Between-treatment difference**  **(Baseline vs week 12)** |
| --- | --- | --- | --- | --- | --- | --- | --- |
|  | **Baseline**  Mean (SD) | **Week 12**  Mean (SD) | **Week 24**  Mean (SD) | **Baseline**  Mean (SD) | **Week 12**  Mean (SD) | **Week 24**  Mean (SD) | **Difference of Least Square Means (95% C.I.), *p*-value** |
| **MMD**  *Change from baseline* | 8·74 (2·67)  - | 3·96 (2·52)  -4·77 (2·68) | 8·72 (3·46)  -0·01 (3·82) | 9·04 (2·73)  - | 8·77 (4·38)  -0·27 (4·75) | 3·93 (2·33)  -5·19 (2·55) | -4·70 (-6·83, -2·58)  <0·0001 |
| **Duration of migraine attacks**  *Change from baseline* | 18·17 (10·55)  - | 9·26 (6·06)  -8·91 (10·10) | 16·90 (14·21)  -1·28 (15·35) | 14·21 (9·62)  - | 15·24 (11·19)  1·03 (9·99) | 7·92 (5·44)  -6·70 (9·56) | -7·57 (-12·62, -2·52)  0·0042 |
| **MHD**  *Change from baseline* | 9·71 (2·75)  - | 4·74 (2·79)  -4·96 (2·68) | 9·19 (4·12)  -0·51 (3·93) | 9·40 (2·99)  - | 8·90 (4·35)  -0·50 (4·77) | 4·33 (2·46)  -5·17 (3·29) | -4·28 (-6·42, -2·14)  0·0002 |
| **MAT**  *Change from baseline* | 7·27 (2·98)  - | 3·32 (2·44)  -3·95 (3·06) | 7·76 (3·21)  0·48 (4·05) | 7·18 (2·74)  - | 8·32 (4·35)  1·14 (4·40) | 3·40 (2·30)  -3·82 (3·07) | -5·04 (-7·10, -2·97)  <0·0001 |
| **Number of days with photophobia**  *Change from baseline* | 5·16 (3·52)  - | 1·62 (1·89)  -3·55 (3·42) | 4·17 (3·01)  -1·00 (3·73) | 5·30 (3·35)  - | 5·18 (4·30)  -0·11 (4·02) | 2·38 (2·15)  -2·82 (3·30) | -3·51 (-5·40, -1·63)  0·0005 |
| **Number of days with phonophobia**  *Change from baseline* | 4·92 (4·00)  - | 1·06 (1·79)  -3·86 (3·53) | 4·36 (2·69)  -0·56 (3·34) | 5·85 (3·19)  - | 5·58 (4·53)  -0·27 (3·96) | 2·46 (2·14)  -3·32 (3·06) | -4·13 (-6·06, -2·20)  <0·0001 |
| **Number of days with nausea**  *Change from baseline* | 4·54 (2·80)  - | 1·91 (2·03)  -2·63 (3·37) | 5·98 (3·90)  1·44 (4·05) | 3·95 (3·57)  - | 3·11 (3·43)  -1·03 (3·68) | 1·07 (1·43)  -2·73 (3·35) | -1·31 (-2·98, 0·35)  0·1190 |
| **Number of days with aura**  *Change from baseline* | 0·64 (1·53)  - | 0·21 (0·61)  -0·43 (1·40) | 0·56 (1·17)  -0·07 (0·91) | 0·72 (1·40)  - | 0·05 (0·25)  -0·70 (1·33) | 0·04 (0·20)  -0·71 (1·46) | 0·17 (-0·10, 0·44)  0·2138 |
| **NRS scores**  *Change from baseline* | 6·55 (1·13)  - | 5·65 (1·97)  -0·90 (1·57) | 6·56 (1·28)  0·02 (1·23) | 6·90 (1·14)  - | 7·12 (1·52)  0·17 (1·16) | 6·05 (2·26)  -0·69 (2·12) | -1·08 (-1·95, -0·20)  0·0169 |
| **HIT-6 scores**  *Change from baseline* | 64·68 (3·62)  - | 51·50 (9·69)  -13·18 (9·53) | 62·59 (5·42)  -2·09 (5·14) | 66·55 (5·62)  - | 62·55 (6·24)  -4·00 (4·49) | 58·29 (7·40)  -7·71 (7·18) | -9·66 (-14·28, -5·04)  0·0001 |
| **ASC-12 scores**  *Change from baseline* | 6·14 (5·02)  - | 3·41 (3·90)  -2·73 (4·66) | 5·09 (5·23)  -1·05 (5·13) | 5·77 (3·99)  - | 6·14 (3·94)  0.·36 (3·85) | 5·24 (4·68)  -0·67 (4·21) | -2·88 (-4·98, -0·78)  0·0083 |
| **HAD-A scores**  *Change from baseline* | 6·41 (3·86)  - | 3·77 (2·81)  -2·64 (2·52) | 5·09 (3·62)  -1·32 (2·80) | 5·73 (4·13)  - | 5·09 (2·88)  -0·64 (3·63) | 4·43 (3·68)  -1·14 (3·05) | -1·62 (-2·99, -0·26)  0·0211 |
| **HAD-D scores**  *Change from baseline* | 5·09 (3·37)  - | 2·91 (2·93)  -2·18 (3·40) | 5·27 (3·64)  0·18 (2·99) | 5.·77 (4·26)  - | 4·41 (3·10)  -1·36 (3·39) | 4·10 (4·06)  -1·57 (2·98) | -1·22 (-2·79, 0·36)  0·1277 |

Abbreviations: Allodynia Symptom Checklist (ASC-12); Confidence Interval (C.I.); Hospital Anxiety and Depression Scales (HAD-A and HAD-D); Headache Impact Test-6 (HIT-6); Monthly number of days with use of Acute Treatments (MAT); Monthly Headache Days (MHD); Monthly Migraine Days (MMD); Numerical Rating Scale (NRS).

Duration of migraine attacks was considered in hours.

**Supplementary Table 10.** Regions showing significant resting state (RS) functional connectivity (FC) changes, from baseline to week 12 of treatment, within the erenumab group in the per protocol set (PPS) population (SPM12 full factorial models adjusted for acquisition scanner and propensity score, *p* <0.05, family-wise error [FWE] corrected for multiple comparisons. Results correct for the number of investigated networks using the Bonferroni approach are marked with *).

| **RS FC networks relevant for migraine** | | | | | | | |
| --- | --- | --- | --- | --- | --- | --- | --- |
| **RS networks** | **Finding** | **Region** | **BA** | **t values** | **K_E_** | **MNI coordinates**  **(x y z)** |  |
| L PAG | Increased RS FC | L SMA | 8 | 6·13 | 93 | -8 22 52 |  |
| R PAG | Increased RS FC | L cerebellum crus I | - | 6·83 | 102 | -32 -56 -36 |  |

Abbreviations: BA=Brodmann area; K_E_=cluster extent; L=left; PAG=periaqueductal gray; R=right; SMA=supplementary motor area.

**Supplementary Table 11.** Regions showing significant resting state (RS) functional connectivity (FC) changes, from baseline to week 12 of treatment, within the placebo group in the per protocol set (PPS) population (SPM12 full factorial models adjusted for acquisition scanner and propensity score, *p* <0.05, family-wise error [FWE] corrected for multiple comparisons. Results correct for the number of investigated networks using the Bonferroni approach are marked with *).

| **Main large scale RS FC networks** | | | | | | |
| --- | --- | --- | --- | --- | --- | --- |
| **RS networks** | **Finding** | **Region** | **BA** | **t values** | **K_E_** | **MNI coordinates**  **(x y z)** |
| Auditory | Decreased RS FC | R cerebellum lob VI | - | 6·45 | 119 | 10 -62 -22 |
|  | Increased RS FC | L SMG | 48 | 5·06 | 105 | -62 -44 30 |
|  |  | R SMG | 48 | 5·49 | 94 | 68 -42 32 |
| Primary visual | Decreased RS FC | L STG | 48 | 5·69* | 190 | -48 -8 -10 |
|  |  | L PaCL  R PaCL | 5  5 | 5·26  4·91 | 129 | -4 -44 64  6 -42 66 |
|  |  | L HIPP | 20 | 6·97 | 100 | -32 -6 -18 |
| Secondary visual II | Decreased RS FC | Cerebellum vermis  R lingual gyrus | -  18 | 6·62*  6·50* | 216 | 6 -62 -4  6 -74 -4 |
|  |  | L STG  L insula | 48  48 | 6·23*  5·97* | 329 | -40 -16 10  -46 -6 -2 |
| SN | Decreased RS FC | R calcarine cortex  L calcarine cortex  R lingual gyrus | 17  17  18 | 9·10*  6·35*  5·96* | 686 | 8 -78 4  -8 -80 4  16 -80 0 |
| **RS FC networks relevant for migraine** | | | | | | |
| L PAG | Decreased RS FC | R cerebellum lob IX | - | 5·03 | 87 | 2 -60 -48 |
| L thalamus | Decreased RS FC | L angular gyrus | 7 | 5·91* | 580 | -38 -72 44 |
|  |  | L precuneus | 31 | 5·73 | 132 | -2 -54 20 |
| R hypothalamus | Decreased RS FC | L cuneus  L precuneus | 17  7 | 4·70  4·39 | 94 | -4 -68 26  -4 -66 36 |
| R thalamus | Decreased RS FC | L precuneus  R precuneus | 31  31 | 6·12*  4·98* | 354 | -6 -58 26  4 -60 28 |
|  | Increased RS FC | R SMA/SFG  L SMA | 6  6 | 7·01*  5·45* | 246 | 60 10 64  0 6 56 |

Abbreviations: BA=Brodmann area; K_E_=cluster extent; HIPP=hippocampus; L=left; PaCL=paracentral lobule; PAG=periaqueductal grey; R=right; SFG=superior frontal gyrus; SMA=supplementary motor area. SMG=supramarginal gyrus; SN=salience network; STG=superior temporal gyrus.

**Supplementary Table 12.** Regions showing significant differences of resting state (RS) functional connectivity (FC) changes from baseline to week 12 of treatment between erenumab and placebo groups in the PPS population (SPM12 full factorial models adjusted for acquisition scanner and propensity score, *p*<0.05, family-wise error [FWE] corrected for multiple comparisons. Results correct for the number of investigated networks using the Bonferroni approach are marked with * and results reported at *p*<0.001, uncorrected are marked with ¶).

|  | **Main large scale RS FC networks** | | | | | |
| --- | --- | --- | --- | --- | --- | --- |
| **RS networks** | **Finding** | **Region** | **BA** | **t values** | **K_E_** | **MNI coordinates**  **(x y z)** |
| Primary visual | Erenumab > Placebo | R calcarine cortex | 17 | 4.70 | 119 | 4 -82 6 |
| Cerebellar | Erenumab > Placebo | L precuneus  R precuneus | 7  7 | 3.81¶  3.77¶ | 74 | -2 -64 50  2 -50 46 |
|  | **RS FC networks relevant for migraine** | | | | | |
| R thalamus | Placebo > Erenumab | R SFG | 6 | 5.60* | 327 | 22 2 64 |
| L PAG | Erenumab > Placebo | R cerebellum lob IX | - | 4.18¶ | 72 | 18 -44 -44 |

Abbreviations: BA=Brodmann area; K_E_=cluster extent; L=left; PAG=periaqueductal gray; R=right; SFG=superior frontal gyrus.

**Supplementary Table 13.** Summary of patients with treatment emergent adverse events.

|  | Erenumab n (%) | Placebo n (%) |
| --- | --- | --- |
| Number of patients with at least one TEAEs | 28 (47.46) | 17 (29.82) |
| Number of patients with at least one treatment-related TEAEs | 16 (27.12) | 5 (8.77) |
| Number of patients with at least one serious TEAEs | 1 (1.69) | 0 |
| Number of patients with at least one TEAEs leading to study discontinuation | 2 (3.39) | 0 |
| Number of patients with at least one TEAEs of special interest | 0 | 0 |
| Number of patients with at least one fatal TEAEs | 0 | 0 |

Abbreviations: TEAEs =Treatment emergent adverse events

A treatment emergent adverse event was defined as an adverse event with a start date during the on-treatment period.

Patients who experienced more than one TEAE were counted only once in each row and in each treatment.

**
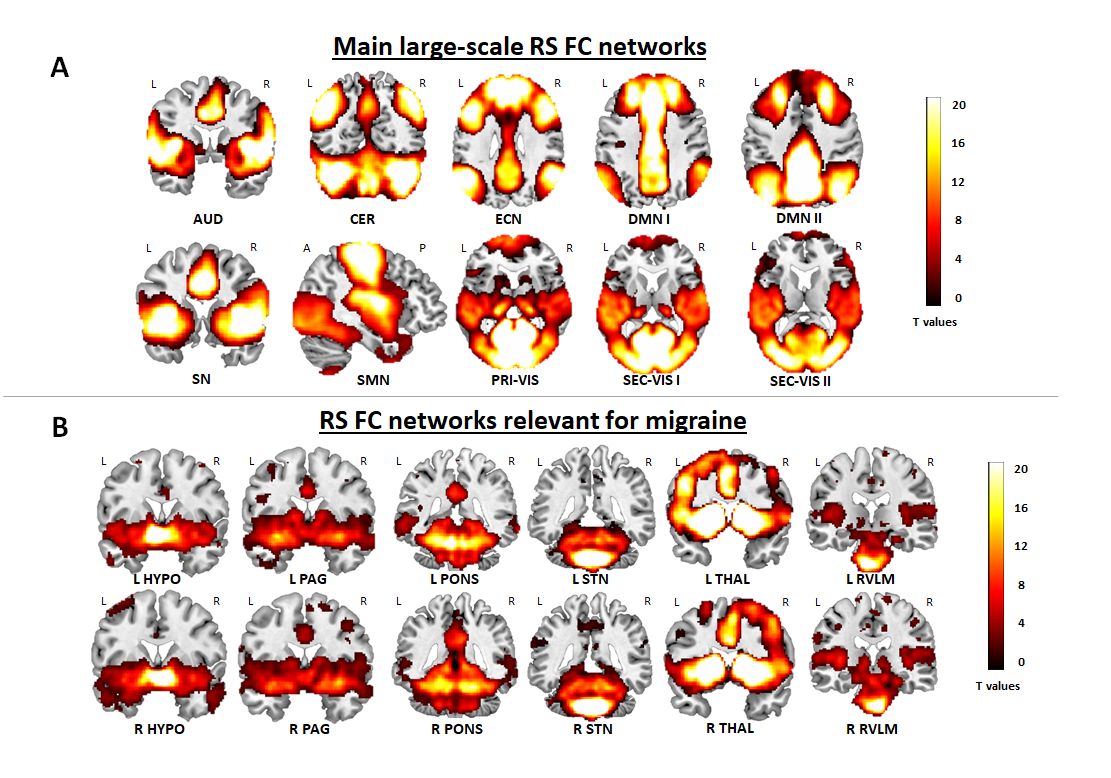
**

**Supplementary Figure 1.** Average spatial maps, derived from all study subjects, of resting state (RS) functional connectivity (FC) networks analyzed in this study (scanner-adjusted one sample t-tests, p<0.05 family-wise error [FWE] corrected). For a better figure readability, networks were divided into: A) main large-scale RS FC networks of the brain; and B) RS FC networks relevant for migraine. Images are in neurological convention. Abbreviations: A=anterior; AUD=auditory network; CER=cerebellar network; DMN=default-mode network; ECN=executive control network; HYPO=hypothalamus; L=left; P=posterior; PAG= periaqueductal gray; PRI-VIS=primary visual network; R=right; RVLM=rostro ventrolateral medulla; SEC- VIS=secondary visual network; SMN=sensorimotor network; SN=salience network; STN=spinal trigeminal nucleus; THAL=thalamus.

**
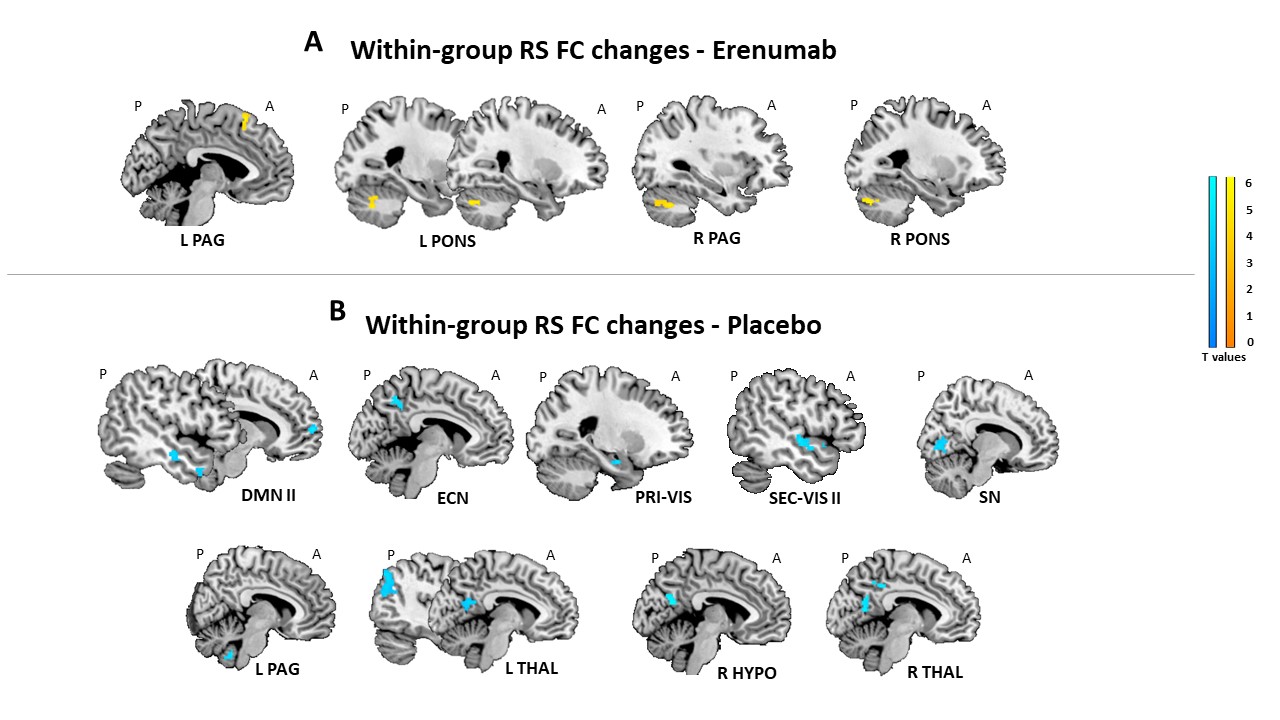
**

**Supplementary Figure 2.** Within-group changes from baseline to week 12 of resting state (RS) functional connectivity (FC) in the erenumab (A) and placebo (B) groups in the full analysis set (FAS) population (propensity score- and scanner-adjusted full factorial models, *p* <0.05, family-wise error [FWE] corrected for multiple comparisons). Increased RS FC is color coded in orange-yellow, while decreased RS FC is color-coded in blue-light. Images are in neurological convention.

Abbreviations: A=anterior; DMN=default-mode network; ECN=executive control network; HYPO=hypothalamus; L=left; PAG=periaqueductal gray; P=posterior; PRI-VIS=primary visual network; R=right; SEC-VIS=secondary visual network; SN=salience network; THAL=thalamus.

**
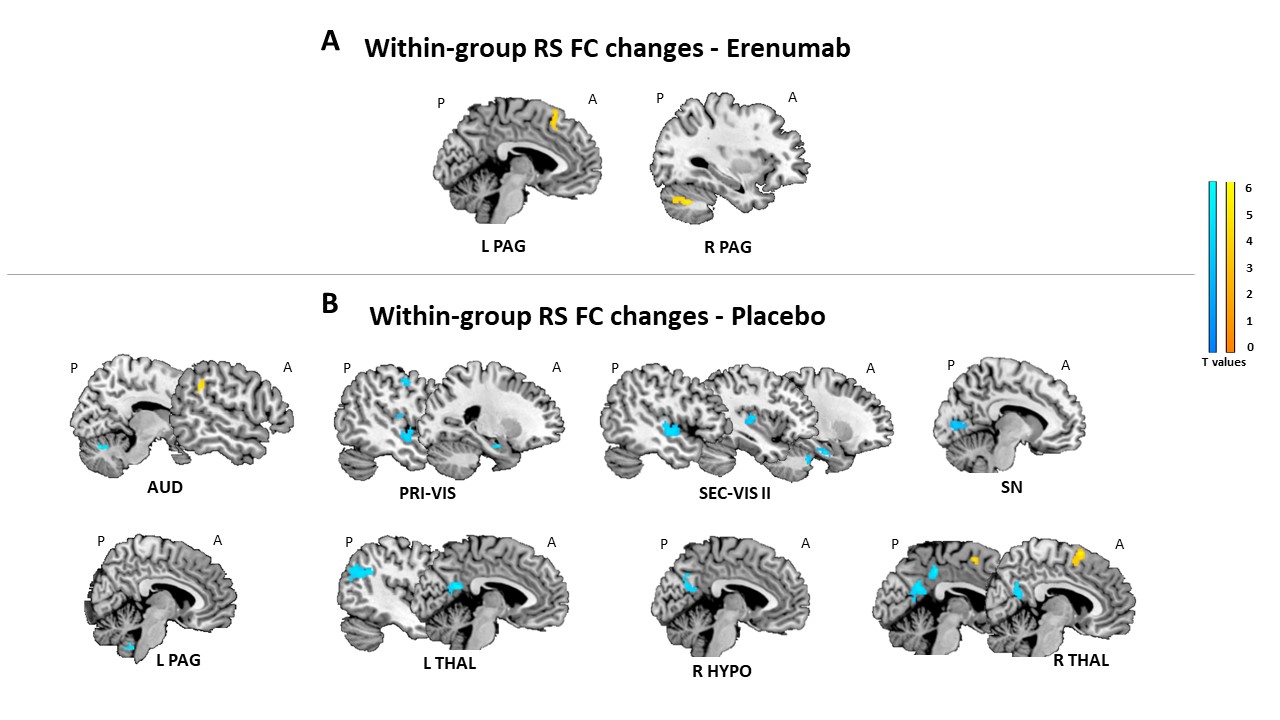
**

**Supplementary Figure 3.** Within-group changes from baseline to week 12 of resting state (RS) functional connectivity (FC) in the erenumab (A) and placebo (B) groups in the per protocol set (PPS) population (propensity score- and scanner-adjusted full factorial models, *p* <0.05, family-wise error [FWE] corrected for multiple comparisons). Increased RS FC is color coded in orange-yellow, while decreased RS FC is color-coded in blue-light. Images are in neurological convention.

Abbreviations: AUD=auditory network; A=anterior; HYPO=hypothalamus; L=left; P=posterior; PAG= periaqueductal gray; PRI-VIS=primary visual network; R=right; SEC-VIS=secondary visual network; SN=salience network; THAL=thalamus.


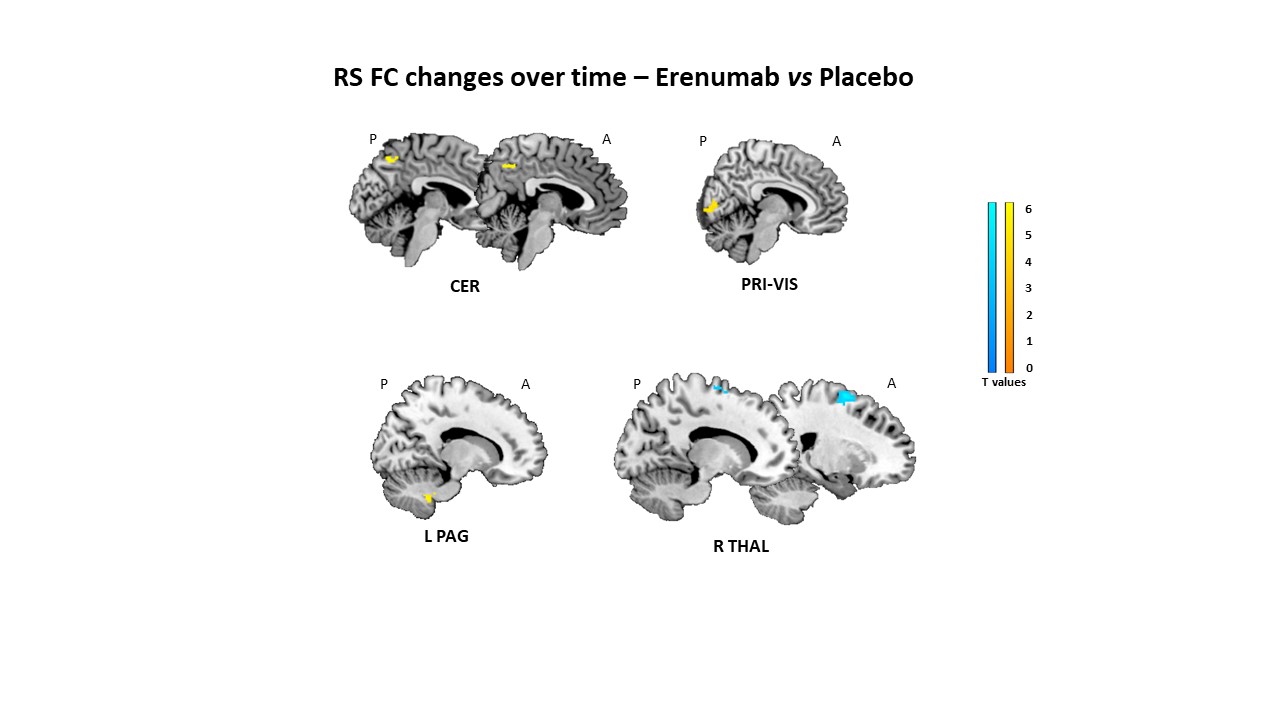


**Supplementary Figure 4.** Significant differences in changes from baseline to week 12 of resting state (RS) functional connectivity (FC) in the erenumab *vs* placebo groups in the per protocol set (PPS) population (propensity score- and scanner-adjusted full factorial models, *p* <0.05, family-wise error [FWE] corrected for multiple comparisons). Increased RS FC in the erenumab *vs* placebo group is color coded in orange-yellow, while decreased RS FC in the erenumab *vs* placebo group is color-coded in blue-light. Images are in neurological convention. Abbreviations: A=anterior; CER=cerebellar network; L=left; P=posterior; PAG=periaqueductal gray; PRI-VIS=primary visual network; R=right; THAL=thalamus.
